# Negative emotional processing and decision-making inhibition deficits in adolescents with NSSI: a cross-sectional ERP study

**DOI:** 10.3389/fpsyg.2026.1905635

**Published:** 2026-08-21

**Authors:** Tingfeng Yu, Keji Wang, Yun Xu

**Affiliations:** 1 Department of Psychology, Chengdu Medical College, Chengdu, China; 2 Sichuan Research Center of Applied Psychology, Chengdu, China

**Keywords:** adolescents, event-related potentials, inhibitory control, negative emotion processing, non-suicidal self-injury

## Abstract

**Background:**

This study utilises ERP technology and the Iowa Gambling Task (IGT) to compare the behavioral and EEG characteristics of adolescents with non-suicidal self-injury (NSSI) and those with depression in response to negative emotional stimuli, with the aim of exploring the specific neural mechanisms underlying negative emotion processing and inhibitory control in adolescents with NSSI.

**Methods:**

This study employed a cross-sectional between-groups design to compare 32 adolescents with NSSI and 34 adolescents with depression. All participants completed the emotion-face-based modified Iowa Gambling Task, whilst 64-channel EEG recordings were simultaneously acquired. The analysis focused on behavioral measures and ERP components such as the early posterior component and LPP.

**Results:**

The net IGT score in the NSSI group was significantly lower than that in the Depression group [*F*(1, 64) = 9.06, *p* = 0.004], and the Group × Emotion interaction was significant (*p* = 0.030), with the largest difference observed under the anger condition. ERP analyses revealed a significant interaction: for the early posterior component (mean amplitude, 140–190 ms), the fear amplitude was significantly more negative (i.e., enhanced) in the Depression group, whilst there was no difference in the NSSI group; for the LPP (mean amplitude, 650–800 ms), the fear amplitude was significantly higher than the neutral amplitude in the NSSI group, whilst there was no difference in the Depression group.

**Conclusion:**

Adolescents with NSSI exhibit a ‘differentiation between early and late stages’ in the processing of negative emotions: in the early stage, there is insufficient differentiation in the encoding of negative emotions, whilst in the late stage, there is a marked attentional bias toward fearful faces; this provides electrophysiological evidence for NSSI.

## 1 Introduction

Non-suicidal self-injury (NSSI) is a common, deliberate form of self-harm among adolescents, with a global prevalence of 15.9–20.5% and as high as 27.4% in China (Poon et al., 2019; Jie et al., 2024). This age range (16–25 years) is consistent with prior event-related potential (ERP) studies in this research area (Zhao et al., 2023; Liang et al., 2025), and we therefore retain the term “adolescents” throughout the manuscript to describe our sample. Although this behavior deviates from social norms (Kiekens and Claes, 2020), it is a significant trigger for suicidal behavior and a risk predictor (Hamza et al., 2012; Tsypes et al., 2018). Its repetitive nature and the difficulty in controlling it make it a key precursor to suicide attempts (Guan et al., 2012). Therefore, a thorough understanding of the neural mechanisms underlying NSSI is crucial for the early identification and prevention of suicide risk.

NSSI is associated with a variety of negative states; individuals often experience high levels of negative emotions such as depression and anxiety prior to self-harming (Tsypes et al., 2018), and approximately 41.6% of adolescents with major depression exhibit NSSI (Nock et al., 2006). Those experiencing higher levels of negative emotions are more likely to report a history of NSSI (Hasking et al., 2018). Such individuals often struggle with emotional regulation and have poor interpersonal relationships (Taylor et al., 2019), whilst poor emotional regulation is frequently associated with biases in the recognition of emotional facial expressions (Schlegel et al., 2020). Research indicates that university students who engage in NSSI recognise negative facial expressions at lower emotional intensities (Ziebell et al., 2020); hospitalised female adolescents with NSSI rated neutral and happy expressions more negatively and exhibited higher arousal in response to negative expressions (In-Albon et al., 2015), suggesting specific alterations in negative emotion processing among adolescents with NSSI.

Negative emotion processing refers to an individual’s perception, evaluation and regulation of negative stimuli. According to Gross’s model of emotional regulation, this involves early stages of automatic attention and later stages of cognitive regulation. According to Gross’s process model of emotion regulation, emotion regulation comprises five core strategiessituation selection, situation modification, attentional deployment, cognitive change, and response modulationoperating across early automatic and late cognitive regulation stages (Gross and John, 2003). Empirical studies have validated this model through both behavioral and neuroimaging evidence, demonstrating that different regulation strategies recruit distinct neural circuits and have differential effects on emotional outcomes (Ochsner et al., 2002).

Decision-making inhibition refers to the capacity to suppress immediate reward impulses in favor of long-term gains in uncertain, emotionally-charged contexts—distinct from response inhibition (withholding motor responses) and primarily assessed by the IGT (Bechara et al., 1994). According to the somatic marker hypothesis, effective decision-making relies on emotional biasing signals integrated in the ventromedial prefrontal cortex to guide choices that are beneficial in the long run. The IGT captures this process by requiring participants to learn, through trial and error, which options yield net gains over time, despite the allure of immediate high rewards. Poor performance on this task thus reflects a decision-making inhibition deficit, characterized by heightened sensitivity to immediate rewards at the expense of long-term outcomes (Bechara et al., 1994).

The interaction between negative emotion processing and decision-making inhibition is key to understanding NSSI. The cognitive-emotional model of NSSI further posits that self-injurious behavior arises from altered estimates of gains versus losses, reflecting underlying decision-making abnormalities (Hasking et al., 2017). These frameworks support that decision-making inhibition deficits may represent a core mechanism underlying NSSI persistence in adolescents.

Event-related potential (ERP) technology can capture brain activity at specific time points with high precision. Previous studies have shown that negative emotions elicit a series of core ERP components. Despite these contributions, two critical gaps remain. First, most ERP studies have predominantly used Go/No-go or oddball paradigms to measure response inhibition (i.e., withholding a motor response; Zhao et al., 2023, 2025). However, decision-making inhibition remains largely unexplored in NSSI adolescents. The IGT (Bao et al., 2024; Nancekivell et al., 2024), which provides a net score indexing the balance between advantageous and disadvantageous choices, serves as a validated behavioral measure of this capacity (Schatten et al., 2015). Second, the interaction between the temporal dynamics of emotional processing (early encoding vs. late attention) and decision-making inhibition has not been systematically investigated. Therefore, the present study employs a modified emotional IGT combined with ERP to examine how early (the early posterior component,140–190 ms) and late (LPP) stages of negative face processing relate to decision-making deficits in NSSI adolescents, compared to a depression-only group.

The IGT indirectly assesses an individual’s ability to inhibit impulsive choices and adhere to rational decision-making by simulating the trade-off between ‘immediate short-term gains’ and ‘long-term overall gains’ (Bechara et al., 1994). Risk-taking behavior, as a key aspect of impulsivity, is particularly relevant to NSSI (Fox et al., 2015). Therefore, the use of the IGT allows for the examination of inhibitory control functions in adolescents with NSSI from the perspective of decision-making inhibition.

Existing research has begun to illuminate the neural underpinnings of emotional and cognitive processing in NSSI. Several recent studies have directly compared depressed adolescents with and without NSSI using ERP paradigms. Zhao et al. (2023) found that depressed adolescents with NSSI exhibited altered N250, P300 and LPP components during a two-choice oddball task with emotional faces, suggesting abnormalities in inhibitory control and emotional processing 40. Liang et al. (2025) further demonstrated group differences in emotional face processing, with NSSI adolescents showing reduced accuracy in recognizing facial expressions and distinct ERP patterns, and suggested that the LPP may serve as a potential neurophysiological marker for identifying NSSI behavior among depressed adolescents (Liang et al., 2025). However, despite these contributions, two critical gaps remain. First, most existing ERP studies have predominantly employed Go/No-go or dual-choice oddball paradigms, which primarily measure response inhibition (i.e., the ability to withhold a prepotent motor response). In contrast, decision-making inhibition has received relatively little attention in NSSI research. The IGT captures aspects of decision-making that are separable from executive functions and general intelligence (Toplak et al., 2010), yet its application in NSSI populations has largely focused on feedback-related neural activity (e.g., the feedback-related negativity, FRN) rather than on the interaction between emotional context and decision-making inhibition per se (Bao et al., 2024). Notably, a recent meta-analysis confirmed that individuals with NSSI exhibit consistent impairments in value-based decision-making, with adolescents showing worse performance characterized by increased reward sensitivity and preference for immediate rewards compared to other age groups (Jiang et al., 2025). Second, the interaction between the temporal dynamics of emotional processing (early encoding vs. late attentional allocation) and decision-making inhibition has not been systematically investigated, as most studies have examined these processes in isolation. Therefore, the present study employs a modified emotional IGT combined with ERP to examine how early (the early posterior component, 140–190 ms) and late (LPP) stages of negative face processing relate to decision-making deficits in NSSI adolescents, compared to a depression-only group. This approach allows us to concurrently probe the temporal dynamics of emotional processing and their impact on decision-making inhibition, providing electrophysiological evidence to support the identification of individuals at high risk of NSSI.

Based on the above analysis, this study proposes the following hypotheses: (H1) During the early encoding stage of negative facial emotion processing, the early posterior component amplitude in the NSSI group and the Depression group will exhibit different patterns of emotional regulation, manifested as a significant group × emotion interaction; (H2) During the late attentional allocation stage, the NSSI group will exhibit a stronger positive LPP bias toward negative (relative to neutral) emotional faces, i.e., a significant group × emotion interaction; (H3) In terms of decision-making inhibition, the NSSI group’s net score on the modified IGT will be lower than that of the Depression group, suggesting that they find it more difficult to inhibit impulsive preferences for options that are disadvantageous in the long term in ambiguous decision-making situations.

## 2 Materials and methods

### 2.1 Subject of the study

This study has been approved by the Ethics Committee, and written informed consent was obtained from all adolescents and their carers. Using G*Power, with a medium effect size of *f* = 0.25, a significance level of *α* = 0.05, and a statistical power of *1-β* = 0.80, the minimum required sample size for this 2 × 4 mixed-design experiment was calculated to be 24 participants. The experiment ultimately included 34 participants in the Depression group and 32 in the NSSI group, meeting the statistical power requirements. The Depression group (*n* = 34) had a mean age of 19.3 years (*SD* = 3.25, 16–25), comprising 18 males and 16 females; the NSSI group (*n* = 32) had a mean age of 18.65 years (*SD* = 2.88, 16–25), comprising 15 males and 17 females. We conceptualize the 16–25 age range as representing a psychosocial adolescent period that retains the core psychological features of adolescence, based on both Arnett’s emerging adulthood theory and the specific developmental patterns observed in East Asian populations. There were no significant differences between the two groups in terms of age [*t*(64) = 0.90, *p* = 0.371] or gender composition [*χ*^2^(1) = 0.37, *p* = 0.546], and the groups were well matched. The results of the independent samples *t*-test showed that the group of adolescents with NSSI behavior (*n* = 32) had a mean score of 31.1 (*SD* = 5.02), and there was no significant difference in the Beck Depression Inventory-II (BDI-II) scores compared with the Depression group (*n* = 34), which had a mean score of 30.6 [*SD* = 4.87; *t*(64) = −0.44, *p* = 0.662 > 0.05]. This result indicates that the severity of depressive symptoms was well matched between the two groups at baseline, thereby ruling out any confounding influence of differences in depressive symptom severity on subsequent experimental measures.

Inclusion criteria for the NSSI group: (1) aged 16–25 years; (2) Meets the clinical diagnostic criteria for NSSI as set out in the Diagnostic and Statistical Manual of Mental Disorders, Fifth Edition (DSM-5); (3) meeting the clinical criteria for depression (BDI-II ≥ 29); (4) Self-injury frequency of ≥4 times per month within the past 3 months (SASI ≥4 times per month); (5) Right-handed, with normal vision and hearing or corrected to normal levels; (6) Willing to sign an informed consent form and participate in subsequent experimental studies.

Inclusion criteria for the Depression group: (1) met the clinical criteria for depression (BDI-II ≥ 29); (2) self-injurious behavior ≤1 episode per year (SASI ≤ 1 episode per year); (3) right-handed, with normal vision and hearing or vision and hearing corrected to normal levels; (4) voluntarily signed an informed consent form. All other criteria were the same as for the NSSI group.

All patients were assessed by two experienced psychiatrists and psychologists and met the inclusion criteria. Exclusion criteria included: (1) diagnosis of any mental disorder other than depression; (2) chronic or severe physical illnesses such as head trauma, epilepsy, thyroid disorders, autoimmune diseases or cardiopulmonary diseases; (3) participants at high risk of suicide; (4) participants unable to complete the EEG experimental tasks.

Medication use was systematically documented for each participant. All participants in both groups (Depression group: 34/34, 100%; NSSI group: 32/32, 100%) were receiving standard antidepressant medication, specifically selective serotonin reuptake inhibitors (SSRIs; including sertraline, escitalopram, and fluoxetine), with stable dosages maintained for at least 4 weeks prior to participation.

### 2.2 Research tools

#### 2.2.1 Questionnaires

All participants completed the Beck Depression Inventory-II (BDI-II) prior to the experiment to assess the severity of depressive symptoms; this scale uses a 4-point scoring system and has good reliability and validity.

The criteria for excluding invalid questionnaires are as follows: (1) a response omission rate of 5% or higher; (2) careless completion, with a single option selected for every item; (3) responses falling outside three standard deviations.

#### 2.2.2 Experimental materials

The facial affect images used in this study were sourced from the Chinese Facial Affective Picture System (CFAPS) and included images depicting neutral emotions and negative emotions (anger, fear, and disgust). The experimenters randomly selected 140 images from the CFAPS database; these images were matched for valence and arousal, and had the same dimensions and resolution (260 × 300, 100 pixels per inch). Images are pre-matched for low-level visual features including brightness, contrast, spatial frequency, and background luminance. These physical properties have been controlled by the database developers through standardized acquisition and post-processing procedures, thereby minimizing potential confounding effects on early visual ERP components. Thirty adolescent participants aged 16–25 (14 males and 16 females, mean age 22.3 years, standard deviation 2.49) were recruited from a hospital to rate the valence and arousal of the images. The data were analysed using a one-way repeated measures ANOVA for emotional type. The results showed that the main effect of emotional type on arousal was significant [*F*(3,87) = 8.30, *p* < 0.010, *η*^2^ = 0.48], with neutral images exhibiting significantly lower arousal than the other three categories; the main effect of emotional type on valence was also significant [*F*(3,87) = 806.67, *p* < 0.010, *η*^2^ = 0.99], with neutral images scoring significantly higher on valence than the other three categories. This indicates that the images selected by the experimenters are suitable for use as experimental materials in subsequent studies.

### 2.3 Experimental tasks and procedures

#### 2.3.1 The Iowa gambling paradigm

**Figure 1:**
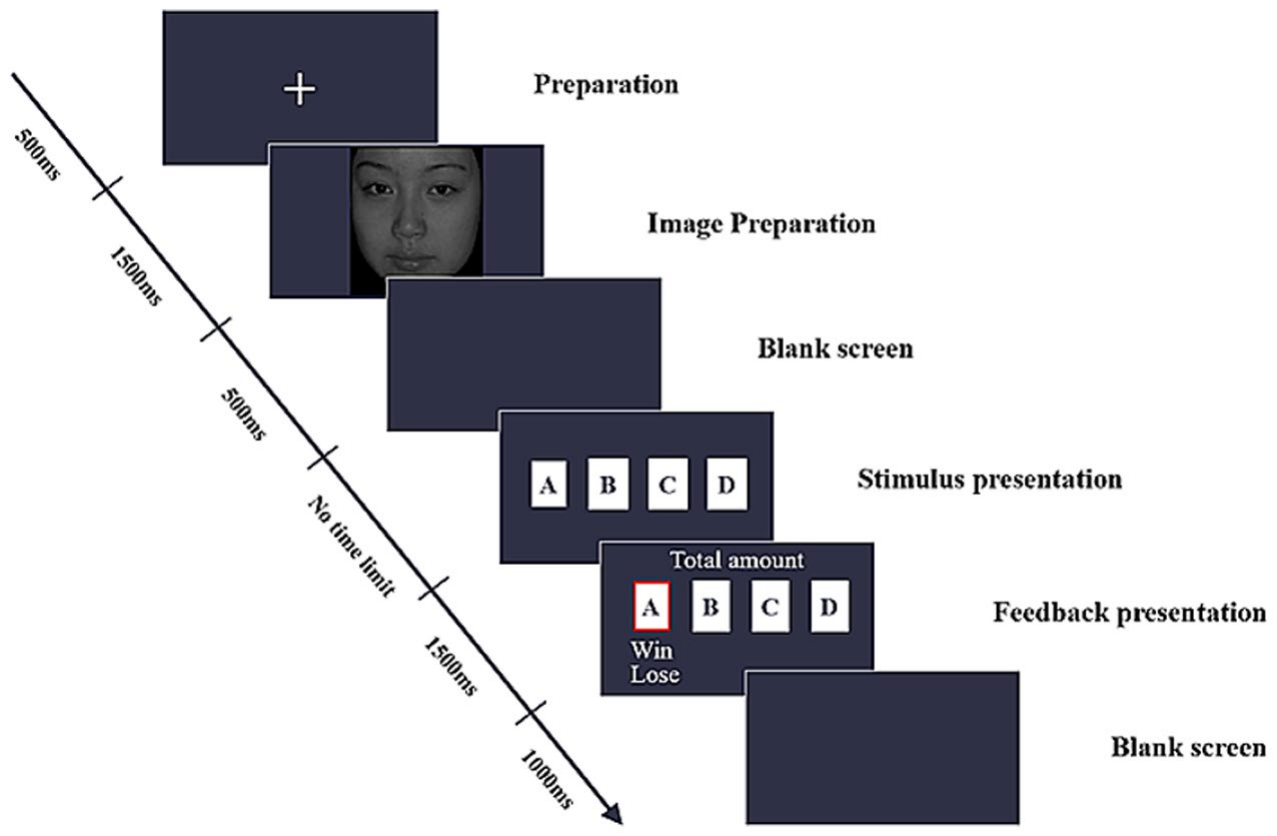
Schematic diagram of the procedure for a single trial in the Modified Iowa Gambling Task (IGT). Each trial sequentially presented a fixation point (500 ms), an emotional face stimulus (1,500 ms), a blank screen (500 ms), a selection phase [unlimited time; the participant pressed a button to select one of four decks: **(A–D)**], feedback (1,500 ms; displaying the profit or loss for that trial and the cumulative amount), and a blank screen (1,000 ms); Instructions are presented before the start of each emotional block.

This study employed a modified IGT based on emotional facial images. A total of four emotional blocks were established (one each for anger, fear, disgust and neutral), with 20 facial images for each emotion presented randomly twice within each block; thus, each emotional block comprised 40 trials (in line with the standard number of trials for the IGT), and the order of presentation of the four emotional blocks was randomised. For statistical analysis, the 40 trials within each emotional block were divided into four equal time-based sub-blocks (Block 1–Block 4, with 10 trials per sub-block), serving as a within-subject ‘sub-block’ factor to examine learning effects during the task. The initial amount was reset at the start of each emotion block (e.g., a virtual principal of $2,000), but the reward and penalty probabilities for the cards remained continuous (i.e., the reward structure of the cards was not reset). The long-term probability of gain for the four decks (A/B/C/D) remained continuous (e.g., Deck A always had an 80% probability of loss, though the amount of a single loss might vary). The physical position of fixed cards remained constant across different emotional blocks (e.g., Card A was always in the top-left corner of the screen), but the reward and punishment sequences for each deck were made pseudo-random via programme control (e.g., ensuring that Card C resulted in a loss 7 times out of every 10 trials). The experimenter first briefs the participant on the experimental procedure and requirements; the participant then completes a personal details form and signs an informed consent form. At the start of the experiment, the following instructions are displayed: “You will first see a series of images depicting different facial expressions. Once the images have disappeared, you will need to complete a card-selection task (the images are unrelated to the value of the cards). You will then be shown four cards labelled A, B, C and D. Your task is to win as many game coins as possible by selecting cards (the initial balance is $2,000); please select one card at a time. Each selection may result in a win or a loss, and your profit or loss will be displayed immediately after each choice. Note: The long-term returns on different cards may vary, so please choose carefully. The higher your total earnings, the greater the actual reward you will receive at the end of the experiment.” This helps participants familiarise themselves with the procedure. During the experiment, behavioral data (card selection outcomes, reaction times, etc.) and EEG data were recorded. The presentation procedure for a single trial is shown in Figure 1.

### 2.4 EEG data acquisition and pre-processing

EEG signals were recorded from 64 scalp electrode sites using a Neuroscan Quick Cap EEG system on a Curry 8 system, used in the current study adopted the international extended 10–20 electrode placement system. Vertical electrooculogram (VEOG) signals were recorded simultaneously from the supraorbital and infraorbital regions of the left eye. Horizontal electrooculogram signal acquisition technology was employed to record signals from the orbital rims of the left and right eyes, respectively. The reference electrode between Cz and CPz was used as the on-line reference point, with a sampling rate set at 1000 Hz and a bandpass range of 0.05–100 Hz. All electrodes required impedance adjustment using conductive gel; data acquisition could only commence once the impedance values were all below 5 kΩ.

Both EEG signal processing and off-line analysis were performed using MATLAB software in conjunction with the EEGLAB toolbox. All EEG data were resampled at 500 Hz and processed using a 0.1–30 Hz bandpass filter, whilst re-referencing was performed based on the average of the M1 and M2 electrodes. The epoch duration was set to 200 ms before the stimulus and 1,000 ms after the stimulus (totalling 1,200 ms). The system automatically excludes time periods containing response errors or obvious artefacts. Subsequently, independent component analysis (ICA) was used to remove artefacts, primarily comprising blinks, horizontal eye movements and some electromyographic (EMG) artefacts. For each stimulus condition, the data were segmented and averaged. Finally, baseline correction was performed by subtracting the 200-millisecond pre-stimulus baseline from the post-stimulus waveform.

### 2.5 ERP data analysis

The EEGLAB toolbox was used to record ERP waveforms under various conditions involving neutral facial stimuli and negative facial stimuli (anger, fear, and disgust). Based on previous research, the analysis was conducted using the mean amplitude of the ERP components. The time windows and electrode selections for each component are as follows: the early posterior component (mean amplitude, 140–190 ms, PO7/PO8) and LPP (mean amplitude, 650–800 ms, CPz/Pz). The mean amplitude for each ERP component was calculated as the average voltage within the specified time window at the corresponding electrode clusters, consistent with the time windows established in prior ERP studies of emotional face processing. The time windows for each component were all referenced to the onset of the emotional face stimulus. The selection of time windows and electrode clusters was determined based on previous studies on the characteristics of event-related potentials for emotional faces, as well as observations of the overall average waveforms. For all ERP components, fixed time windows were applied uniformly across all participants and experimental conditions to ensure consistency in amplitude extraction. The selection of these windows was based on both prior literature and visual inspection of the grand-average waveforms. It is important to note that the mean amplitude for the early component at PO7/PO8 was calculated using an average mastoids reference. Given that this reference is non-neutral and can significantly attenuate occipito-temporal activity, and given that the faces in this paradigm served as a task-irrelevant decision background, the activity in this early window is not interpreted as a canonical N170 but rather as an early posterior component reflecting sensory encoding. The selection of time windows and electrode clusters was determined based on previous studies on the characteristics of event-related potentials for emotional faces, as well as observations of the overall average waveforms.

### 2.6 Statistical analysis

The behavioral data in this study employed a 2 (group) × 4 (emotion) × 4 (block) mixed-design, whilst the ERP data utilised a 2 (group) × 4 (emotion) mixed-design. In the analysis of behavioral data, group served as a between-subjects variable (Depression, NSSI), whilst emotion type (neutral, anger, fear, disgust) and block (Block 1–Block 4) served as within-subjects variables. For the analysis of variance (ANOVA) of mean ERP amplitudes, group (Depression, NSSI) was treated as a between-subjects factor, and valence condition (emotion type) as a within-subjects factor. The dependent variables were behavioral measures (net scores) and ERP measures (mean amplitudes of the early posterior component, 140–190 ms, and LPP components). All statistical analyses were conducted using statistical software such as SPSS 23.0 and MATLAB 2025a. Where ANOVA results were significant, post-hoc tests (using Holm’s correction) were performed to clarify the specific patterns of between-group differences. If the sphericity assumption was violated, the Greenhouse–Geisser correction was applied. A *p*-value of <0.050 was used as the criterion for statistical significance in all statistical analyses.

## 3 Results

### 3.1 Behavioral data results

A mixed-design repeated-measures analysis of variance (ANOVA) was conducted on the IGT net scores (difference between reward-seeking and punishment-avoidance choices), with a 2 (group: Depression group, NSSI group) × 4 (emotion: anger, fear, disgust, neutral) × 4 (block: Block 1–Block 4) design, where group was a between-subjects factor and emotion and block were within-subjects factors; Where the sphericity assumption was violated, Greenhouse–Geisser (GG)-corrected *p*-values were reported. Descriptive statistics for IGT net scores across groups under different emotion and block conditions are presented in Table 1, and the results of the ANOVA are shown in Table 2.

**Table 1:** Descriptive statistics of IGT net scores for each emotion and block condition across the two groups of participants [M (SD)].

Group	Emotion	Block 1	Block 2	Block 3	Block 4	Overall mean
Depression group (*n* = 34)	Anger	1.53 (5.12)	1.53 (6.05)	2.59 (4.74)	1.94 (4.74)	1.90 (4.52)
	Fear	0.59 (5.43)	0.71 (5.35)	0.82 (6.44)	0.76 (5.97)	0.72 (4.83)
	Disgust	−0.29 (6.57)	1.06 (6.27)	1.18 (6.27)	1.29 (6.51)	0.81 (5.79)
	Neutral	−1.24 (5.42)	−0.71 (5.61)	0.24 (5.99)	−0.41 (6.34)	−0.53 (5.02)
NSSI group (*n* = 32)	Anger	−3.38 (4.38)	−1.94 (4.08)	−3.50 (4.63)	−3.06 (4.98)	−2.97 (3.27)
	Fear	−1.50 (5.66)	−1.56 (5.65)	−1.38 (5.93)	−1.44 (5.93)	−1.47 (4.89)
	Disgust	−2.56 (4.52)	−2.12 (4.57)	−1.31 (5.34)	−0.50 (6.05)	−1.62 (4.00)
	Neutral	−2.94 (5.23)	−1.19 (5.83)	−3.38 (5.36)	−1.69 (5.64)	−2.30 (4.63)

**Table 2:** Results of the analysis of variance for the 2 × 4 × 4 factorial design of IGT net score.

Source of variance	df	*F*	*p*	*η*p^2^
Group (A, inter-group)	1, 64	9.06	0.004	0.124
Emotion (B)	3, 192	1.65	0.186	0.025
Group × Emotion	3, 192	3.20	0.030	0.048
Subgroup (C)	3, 192	2.97	0.038	0.044
Group × Subgroup	3, 192	1.61	0.193	0.025
Emotion × Subgroup	9, 576	0.76	0.636	0.012
Group × Emotion × Subgroup	9, 576	0.95	0.475	0.015

Group is a between-subjects factor, and its *p*-values have not been adjusted; mood, block and all interaction terms are repeated-measures (within-subjects) factors; where the sphericity assumption is violated, *p*-values adjusted using the Greenhouse–Geisser method are reported. *df* denotes the degrees of freedom before adjustment, and *η*p^2^ denotes partial *η*^2^.

2Group is a between-subjects factor, and its *p*-values have not been adjusted; mood, block and all interaction terms are repeated-measures (within-subjects) factors; where the sphericity assumption is violated, p-values adjusted using the Greenhouse–Geisser method are reported. df denotes the degrees of freedom before adjustment, and *η*p^2^ denotes partial *η*^2^.

The results of the analysis of variance (Table 2) show that the main effect of group was significant, [*F*(1, 64) = 9.06, *p* = 0.004, *η*p^2^ = 0.124]: the net IGT score for the Depression group was positive overall (M = 0.72), significantly higher than that of the NSSI group (M = −2.09), indicating that the NSSI group was more inclined to make choices that were disadvantageous in the long term during the ambiguous decision-making task. The main effect of block was significant, [*F*(3, 192) = 2.97, *p* = 0.038, *η*p^2^ = 0.044]: net scores generally increased as the task progressed (lowest in Block 1, M = −1.18; Block 4 = −0.35), suggesting that both groups exhibited some learning effect during the task; however, *post hoc* pairwise comparisons between blocks, following Holm correction, did not reach significance (ps > 0.050). The main effect of emotion was not significant, [*F*(3, 192) = 1.65, *p* = 0.186].

There was a significant Group × Emotion interaction, [*F*(3, 192) = 3.20, *p* = 0.030, *η*p^2^ = 0.048]. Simple effects analysis (conducted using independent samples *t*-tests between groups under each emotion condition, with Holm correction) indicated that the between-group differences were primarily driven by the Anger condition: Under the anger condition, the net score for the Depression group (M = 1.90) was significantly higher than that of the NSSI group [(M = −2.97), *t*(64) = 4.98, *p* < 0.001 (Holm-corrected *p* < 0.001), Cohen’s *d* = 1.23]; whereas differences between the two groups under the fear [*t*(64) = 1.83, *p* = 0.072], disgust [*t*(64) = 1.97, *p* = 0.053] and neutral [*t*(64) = 1.48, *p* = 0.143] conditions were not significant after correction. Regarding simple effects within groups, the Depression group’s net score for the anger condition was significantly higher than that for the neutral condition [*t*(33) = 3.19, p_corr = 0.019]; pairwise differences between the remaining emotions were not significant. In the NSSI group, differences between emotion conditions were not significant after Holm correction (ps_corr > 0.150). Furthermore, the Group × Block, Emotion × Block, and Group × Emotion × Block three-way interactions were all non-significant (ps > 0.180).

### 3.2 EEG results

This study systematically examined differences in the average amplitude of two ERP components the early posterior component (140–190 ms) and LPP between the NSSI group and the Depression group under different emotional conditions (anger, fear, disgust and neutral) in the modified IGT task.

Table 3 presents the descriptive statistics for the mean amplitude of the two ERP components (the early posterior component, LPP) across the two groups and under each emotional condition; Table 4 presents the results of a 2 (group) × 4 (emotion) mixed-design analysis of variance, with group as the between-subjects factor and emotional condition as the within-subjects factor.

**Table 3:** Descriptive statistics of the mean amplitudes of the early posterior component (140–190 ms) and LPP for both groups of participants across each emotional condition [M (SD), μV].

Components	Group	Anger	Fear	Disgust	Neutral
The early posterior component (mean amplitude, 140–190 ms)	Depression	2.91 (3.03)	1.63 (2.06)	2.87 (3.40)	2.36 (2.28)
	NSSI	2.43 (2.39)	2.50 (2.30)	2.55 (2.32)	2.21 (2.30)
LPP	Depression	6.00 (10.37)	4.36 (9.62)	6.92 (7.16)	5.80 (8.61)
	NSSI	6.37 (5.72)	6.97 (7.23)	6.48 (5.45)	5.04 (5.89)

**Table 4:** Results of a 2 × 4 mixed-design analysis of variance for the mean amplitude of each ERP component.

Components	Source of variance	df	*F*	*p*	*η*p^2^
The early posterior component (mean amplitude, 140--190 ms)	Group	1, 64	0.00	0.972	<0.001
	Emotion	3, 192	3.38	0.037	0.050
	Group × Emotion	3, 192	3.06	0.046	0.046
LPP	Group	1, 64	0.07	0.798	0.001
	Emotion	3, 192	1.69	0.189	0.026
	Group × Emotion	3, 192	2.92	0.045	0.044

Group is a between-subjects factor, and its *p*-values have not been adjusted; mood and the Group × Mood interaction are repeated-measures (within-subjects) factors; where the sphericity assumption is violated, the *p*-values reported are those adjusted using the Greenhouse–Geisser method. *df* denotes the degrees of freedom before adjustment, and *η*_p_^2^ denotes partial *η*^2^.

#### 3.2.1 Early posterior component (mean amplitude,140–190 ms, PO7/PO8)

**Figure 2:**
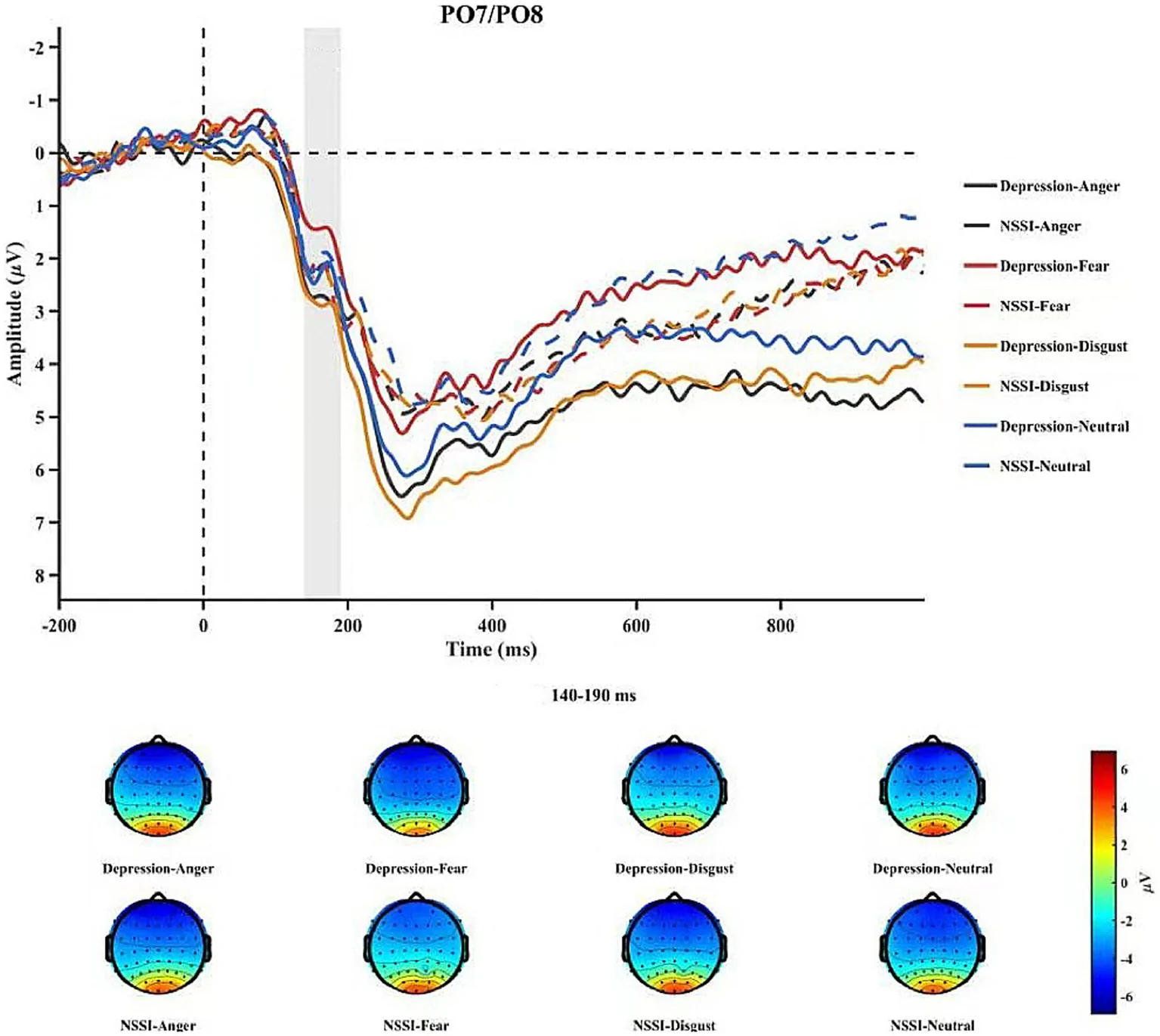
Topography of the overall mean ERP waveforms and the early posterior component (mean amplitude, 140–190 ms) for the PO7/PO8 electrode cluster across the two groups under four emotional conditions. Solid lines represent the Depression group, and dotted lines represent the NSSI group; black denotes anger, red denotes fear, orange denotes disgust, and blue denotes the neutral condition; the grey shading indicates the analysis window for this component. Below are the scalp potential topography maps for both groups within the corresponding time windows for each emotional condition.

A repeated measures analysis of variance revealed a significant main effect of emotion [*F*(3,192) = 3.38, *p* = 0.037, *η*p^2^ = 0.050], and a significant interaction between group and emotion [*F*(3,192) = 3.06, *p* = 0.046, *η*p^2^ = 0.046]. Independent samples *t*-tests comparing the Depression group with the NSSI group across all conditions did not reach significance (*p* > 0.050). Exploratory within-group paired comparisons revealed that the mean early posterior component amplitude for fear in the Depression group was significantly more negative (i.e., enhanced) than that for anger, disgust, and neutral conditions (*p*-values all < 0.050, absolute values of Cohen’s d ranging from 0.39 to 0.43); no significant differences were observed across emotional conditions in the NSSI group (*p* > 0.050). These findings suggest that adolescents with NSSI exhibit a reduced capacity for neural differentiation between different negative emotions during the early stage of early sensory encoding processes compared to the Depression group, whereas the Depression group exhibited a unique pattern of selectively enhanced amplitude exclusively under the fear condition. This suggests that the Depression group may possess specific early neural processing characteristics in response to fearful faces, whereas the early posterior component (140–190 ms) amplitudes in the NSSI group tended to be consistent across all emotional conditions, showing no differentiation in neural responses between emotion types. In the present study, the early ERP activity recorded at the occipito-temporal sites (PO7/PO8) during the 140–190 ms window showed a positive-going deflection rather than the typical negative N170. We attribute this to two main factors: first, the use of an average mastoids reference, which is known to attenuate the N170 originating from nearby temporal areas; and second, the unique nature of our modified IGT paradigm, where faces were task-irrelevant and served as a decision background, which may have reduced the face-specific N170 response. Consequently, we refer to this component as an “early posterior component” and interpret it as reflecting early sensory encoding processes. The waveform waveforms and the EEG topographic maps are presented in Figure 2.

#### 3.2.2 LPP component (mean amplitude, 650–800 ms, CPz/Pz)

**Figure 3:**
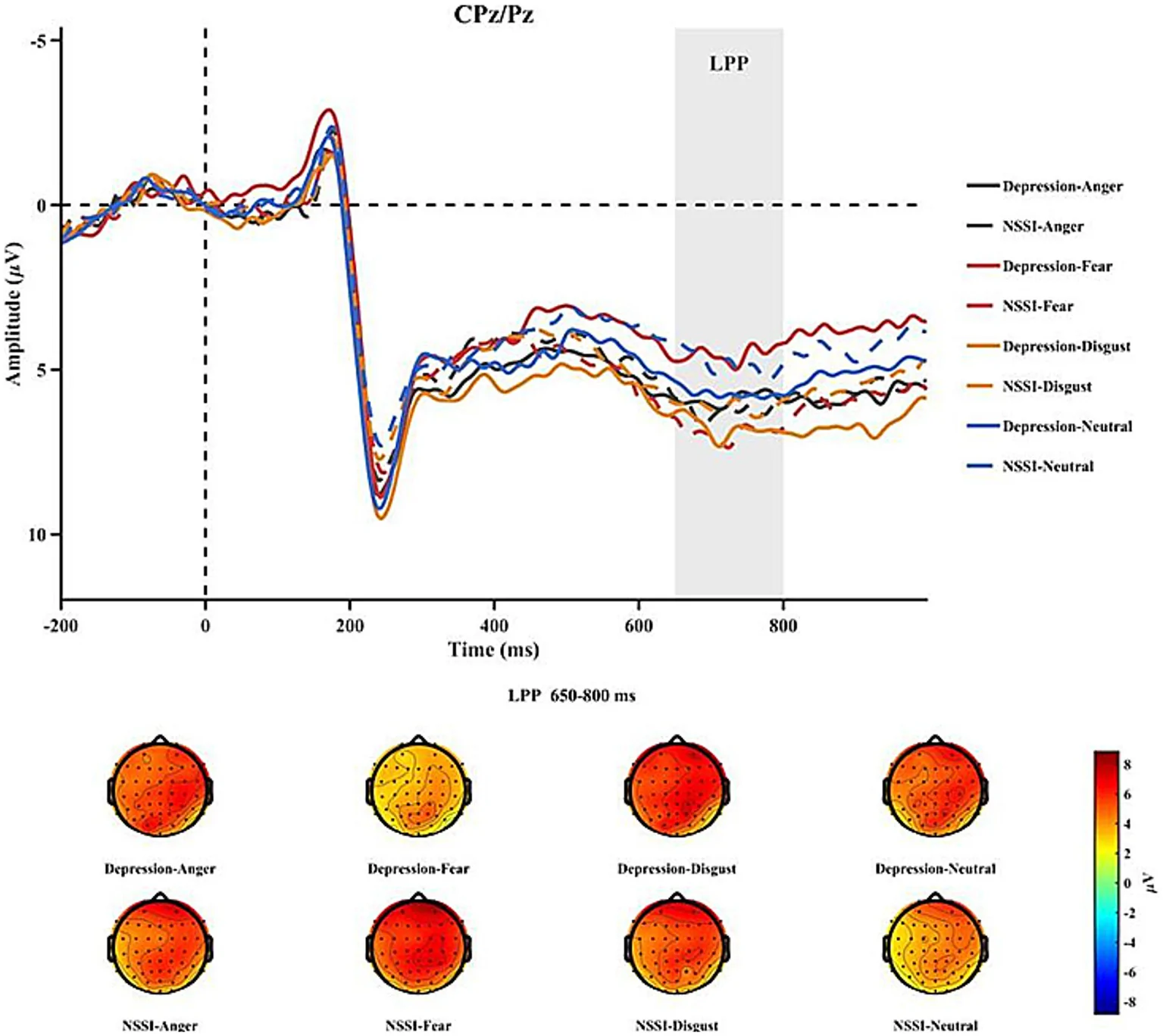
Topographic maps of the overall average ERP waveforms and LPP components (mean amplitude, 650–800 ms) for the CPz/Pz electrode cluster in both groups across the four emotional conditions. Solid lines represent the depression group, and dashed lines represent the NSSI group; black denotes anger, red denotes fear, orange denotes disgust, and blue denotes the neutral condition; the grey shading indicates the analysis window for this component. Below are the scalp potential topography maps for both groups within the corresponding time windows for each emotional condition.

**Figure 4:**
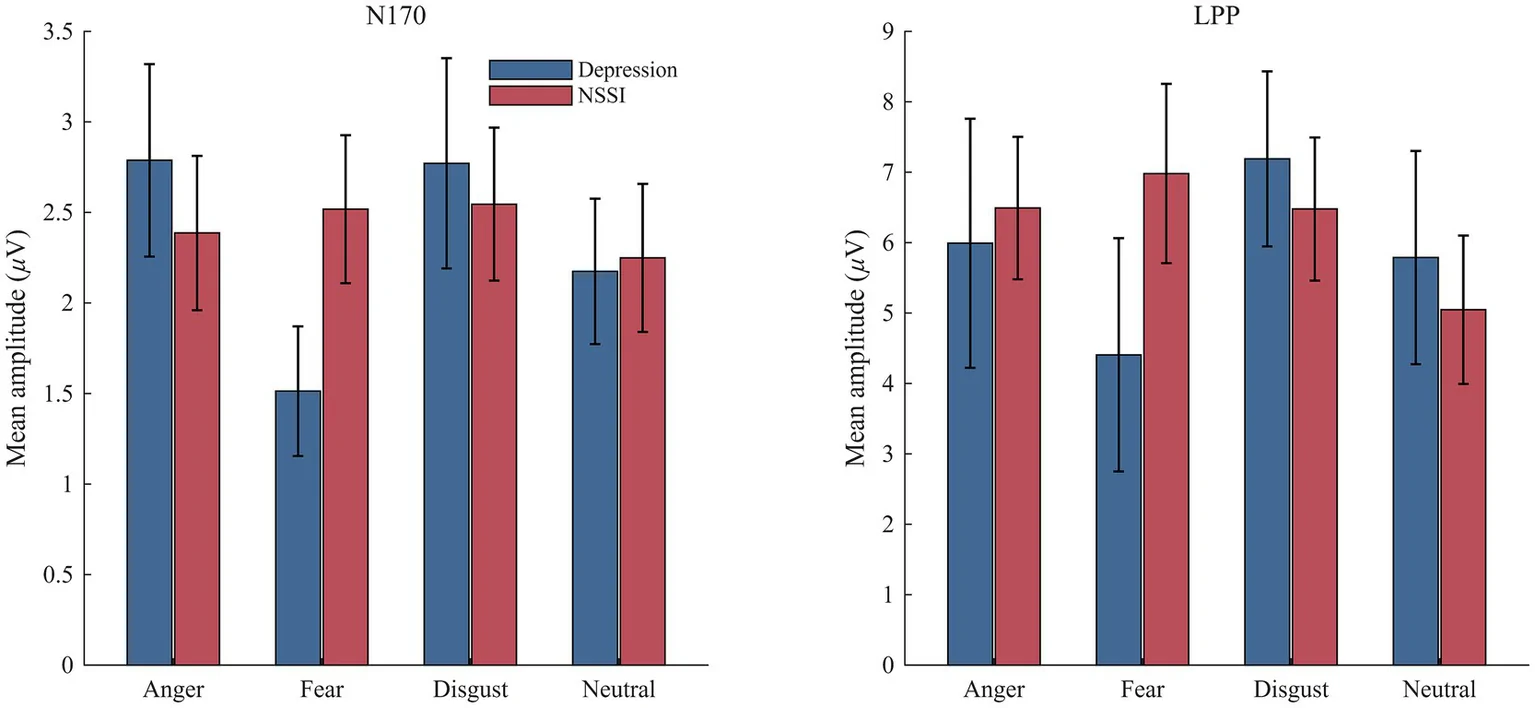
Mean amplitudes (μV) of the **(A)** early posterior component (140–190 ms) and **(B)** LPP components for each emotion condition in the Depression and NSSI groups. Bars indicate M ± SEM (*n* = 66).

**Figure 5:**
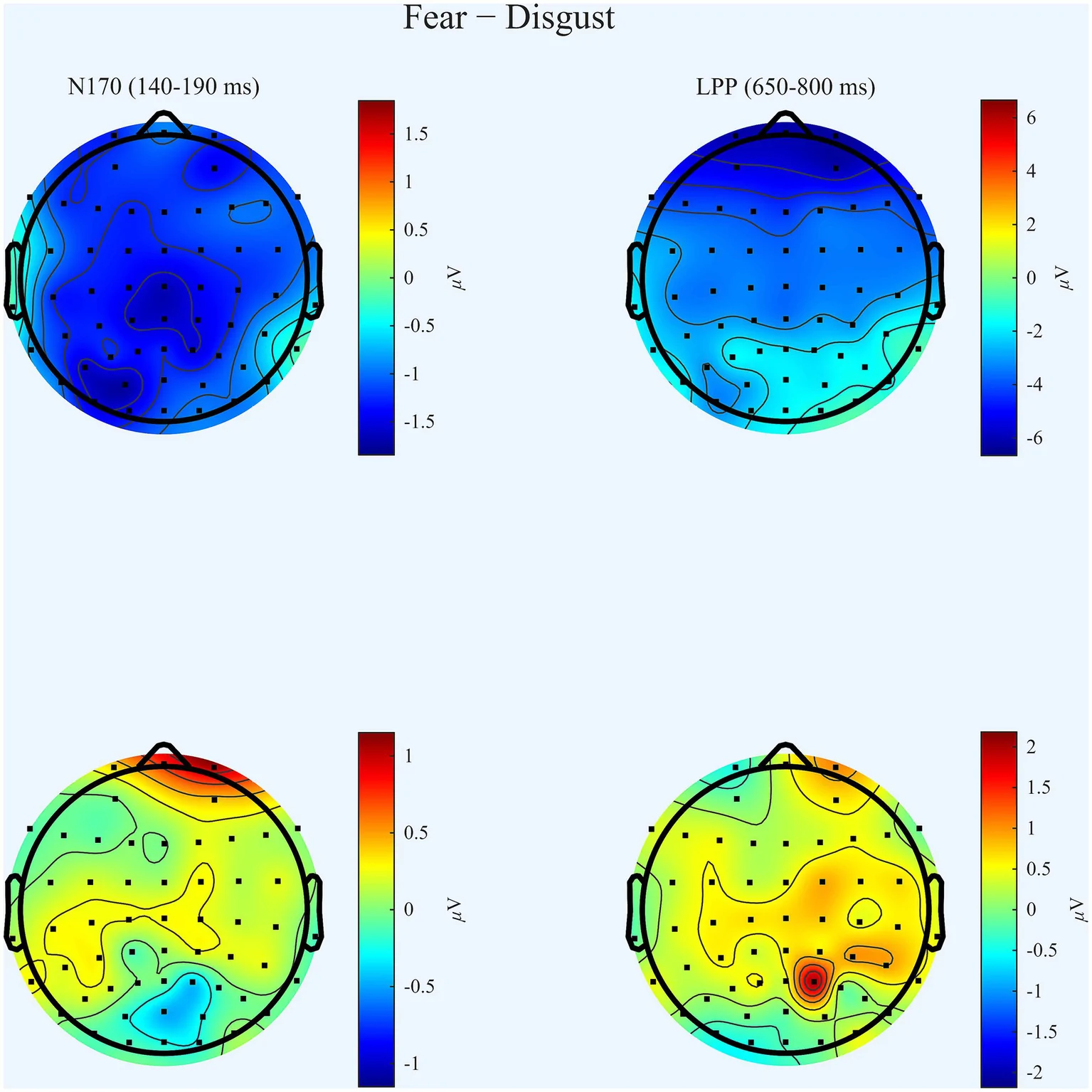
Difference topographies (Fear − Disgust) within the early posterior component (140–190 ms) and LPP (650–800 ms) windows. Top row: Depression group; bottom row: NSSI group. Warm (red) colours indicate a more positive amplitude for Fear.

The mean LPP amplitude did not reveal a significant main effect of group or emotion, but the interaction between group and emotion was significant [*F*(3,192) = 2.92, *p* = 0.045, *η*p^2^ = 0.044]. Differences between the Depression and NSSI groups under all conditions failed to reach significance. Exploratory within-group comparisons revealed that the mean LPP amplitude in the NSSI group was generally higher in negative emotion conditions than in the neutral condition; however, pairwise comparisons indicated that only the Fear condition was significantly higher than the neutral condition [Fear vs. Neutral: *t*(31) = 2.27, *p* = 0.030, dz = 0.40]; the increases in the Anger and Disgust conditions relative to the Neutral condition were consistent in direction but did not reach significance [Anger vs. Neutral: *t*(31) = 1.81, *p* = 0.080, dz = 0.32; Disgust vs. Neutral: *t*(31) = 1.95, *p* = 0.060, dz = 0.35]. Waveforms and topographic maps revealed widespread late positive LPP activity between 650 and 800 ms, with the direction of the Depression–NSSI difference not being entirely consistent across the different emotional conditions. The waveform waveforms and the EEG topographic maps are presented in Figure 3. Mean amplitudes (μV) of the (A) the early posterior component (140–190 ms) and (B) LPP components for each emotion condition in the Depression and NSSI groups in Figure 4. Difference topographies (Fear − Disgust) within the early posterior component (140–190 ms) and LPP (650–800 ms) windows in Figure 5. Top row: Depression group; bottom row: NSSI group. Warm (red) colours indicate a more positive amplitude for Fear.

## 4 Discussion

This study employed a modified IGT combined with event-related potential (ERP) techniques to systematically compare differences in brain electrical activity between adolescents with NSSI and those with depression (without NSSI) under negative facial stimulus conditions, focusing on neurophysiological indicators such as early facial coding (early posterior component,140–190 ms) and late emotional attentional allocation (LPP).

### 4.1 Characteristics of negative emotion processing in adolescents with NSSI

This study found that, during the early encoding phase, there was no significant main effect of group (NSSI vs. Depression) on the early posterior component (140–190 ms), but the interaction between group and emotion was significant:the early posterior component (140–190 ms) amplitude in the Depression group was specifically enhanced (more negative) in response to fear, whereas no significant differentiation was observed across emotions in the NSSI group. This suggests that NSSI adolescents retain their overall ability to encode facial emotions, but experience difficulties in differentiating neural responses to different negative emotions (particularly fear). While this early component is typically associated with structural encoding of faces (the N170), its morphology in our study was influenced by both our referencing scheme (average mastoids) and the task-relevance of the stimuli (Eimer and Holmes, 2007). Therefore, we cautiously interpret this as evidence that NSSI adolescents may show difficulties in the early sensory differentiation of negative emotions, which could represent a vulnerability factor for later attentional biases. However, the atypical nature of this component warrants cautious interpretation and highlights the need for replication in future studies. During the late processing phase, the NSSI group exhibited a negative emotional bias effect: the LPP amplitude elicited by fearful faces was significantly higher than that for neutral faces, with similar trends observed for anger and disgust, whereas no significant differences in LPP were found across emotions in the Depression group. The LPP reflects the brain’s motivational attention and salience tracking of emotional stimuli during the post-perceptual phase; the more salient the stimulus, the larger the LPP (Keifer et al., 2019). Importantly, the LPP is not a direct measure of inhibitory control per se, but rather indexes the allocation of attentional resources to emotionally salient stimuli (Hajcak et al., 2010). Thus, our ERP findings capture the temporal dynamics of emotional processingearly encoding (the early posterior component, 140–190 ms) and late attentional allocation (LPP). The enhanced allocation of attentional resources in the late stage observed in the NSSI group suggests difficulty in maintaining a balance between neutral and negative stimuli, reflecting difficulties in emotional regulation at the electrophysiological level. A recent study of 106 adolescents with depression (with and without NSSI) also found that the LPP can serve as a potential neurophysiological indicator for identifying and preventing NSSI behavior in patients with depression (Politte-Corn et al., 2024) The LPP patterns observed in the NSSI group in this study are highly consistent with the aforementioned findings, supporting the use of LPP as a sensitive probe for emotional processing abnormalities associated with NSSI. In contrast, the LPP in the Depression group showed no significant emotional differences, which may reflect a relatively balanced late attentional allocation or a consistent trend of enhancement or attenuation across different emotions.

### 4.2 Deficits in decision-making inhibition in NSSI adolescents

Our behavioral data revealed that the NSSI group exhibited significantly lower IGT net scores than the Depression group, indicating a pronounced deficit in decision-making inhibition, particularly under the anger condition. Consistent with our findings, recent studies have demonstrated that adolescents with depressive disorders who engage in NSSI exhibit significantly lower IGT net scores compared to those without NSSI, and these lower scores are associated with heightened cognitive impulsivity (Jiang et al., 2024). Similarly, Bao et al. (2024) found that MDD patients with NSSI showed a greater tendency to make risky decisions during the IGT, with their electrophysiological responses to losses versus gains correlated with enhanced impulsivity (Bao et al., 2024). This suggests that negative emotional contextsespecially social threat cues like angerdisrupt the ability of NSSI adolescents to suppress impulsive choices in favor of long-term rational gains.

### 4.3 The interaction between negative emotion processing and decision-making inhibition

Integrating our behavioral and electrophysiological findings, a coherent picture emerges regarding how aberrant emotional processing may compromise decision-making inhibition in NSSI adolescents. Our behavioral data revealed that the NSSI group exhibited significantly lower IGT net scores, particularly under the anger condition, indicating that negative emotional contextsespecially social threat cuesdisrupt their ability to suppress impulsive choices in favor of long-term rational gains (Nancekivell et al., 2024). This aligns with recent evidence demonstrating that adolescents with NSSI show impaired response inhibition when exposed to negative emotional stimuli, as reflected by increased P3d amplitudes and altered oscillatory activity (Zhao et al., 2025). Moreover, neural responses to social decision-making have been shown to correlate with emotion regulation difficulties and personality traits in NSSI adolescents, preliminary confirmation the link between emotional processing and decision-making deficits (Wei et al., 2025).

Critically, our ERP findings illuminate the temporal dynamics underlying this behavioral deficit. At the early encoding stage, the NSSI group exhibited insufficient neural differentiation between negative emotions (as indexed by the early posterior component, 140–190 ms), whereas at the late attentional stage, they demonstrated an exaggerated LPP response to fearful faces. This pattern is consistent with the emotion-cognition interaction model, which posits that negative emotional stimuli activate limbic regions (such as the amygdala), leading to an imbalance in resource allocation to cognitive control areas (such as the prefrontal cortex), thereby reducing the efficiency of inhibitory control (Zhao et al., 2025). Recent studies have similarly reported that adolescents with repetitive NSSI display impaired interference inhibition toward disgusted faces (evidenced by smaller P2d and larger LPCd amplitudes) and general deficits in response inhibition across emotional contexts (reflected by reduced P3d amplitudes; Zheng et al., 2026). These findings suggest that interpersonal stress stimuli—particularly negative facial expressions disrupt both early attentional filtering and later cognitive control processes in NSSI adolescents.

It should be noted, however, that the emotional modulation patterns observed in the early posterior component (140–190 ms) and LPP did not directly correspond to the behavioral IGT findings: whereas the LPP showed a fear-specific enhancement in the NSSI group, the most pronounced group difference in IGT net scores was observed under the anger condition. This discrepancy suggests that the relationship between the temporal dynamics of emotional processing and decision-making inhibition may be more complex than initially hypothesized, and that different negative emotions may exert differential effects on neural processing versus behavioral choice. Future studies with larger sample sizes should conduct correlational analyses between individual ERP emotional modulation indices (e.g., fear-minus-neutral LPP difference) and IGT performance to directly test whether early and late emotional processing abnormalities predict decision-making deficits at the individual level. Our conclusions are based on between-group comparisons of overall means; given the relatively small sample size and the exploratory nature of this cross-sectional study, no individual-level correlation analyses linking ERP emotional regulation to IGT performance were conducted. We therefore interpret our findings as preliminary electrophysiological evidence consistent with the emotion–cognition interaction framework, rather than conclusive proof of a causal pathway.

### 4.4 Limitations of the study and future directions

This study has the following limitations: Firstly, although the interaction effect of ERP components was significant, the between-group comparisons across different emotions did not reach statistical significance. This suggests that the interaction effect primarily stems from differences in emotional patterns within groups rather than stable between-group differences under specific conditions. Future studies could increase the sample size to enhance statistical power; Second, the absence of a healthy control group precludes a direct determination of whether the abnormalities associated with NSSI represent a unique neural feature or a secondary manifestation of comorbid depression. Future studies should include a healthy control group to further clarify the specific mechanisms involved. Finally, although all participants were inpatients receiving standard antidepressant medication, and medication status (type, dosage, and duration) was basically balanced between the two groups, we cannot completely rule out the possibility that SSRIs may have exerted subtle non-specific effects on certain ERP components (e.g., late attentional processes). However, because medication use was entirely matched across groups, such effects are unlikely to account for the observed group differences. Nonetheless, future studies may consider including medication-naïve patients or employing washout designs to further isolate the neural correlates specifically associated with NSSI.

## 5 Conclusion

This study systematically compared the electrophysiological characteristics of adolescents with NSSI and those with depression during the processing of negative emotional faces using the IGT-ERP paradigm. The main conclusions are as follows: (1) Adolescents with NSSI exhibited insufficient differentiation in neural responses to different negative emotions during the early encoding phase (the early posterior component 140–190 ms), whereas during the late attentional allocation phase (LPP), they demonstrated a negative emotional bias predominantly toward fearful faces; (2) Our behavioral finding of lower IGT net scores in the NSSI group supports the utility of the IGT net score as a behavioral index of decision-making inhibition deficits in this population, as it captures the ability to suppress impulsive choices in favor of long-term gains in emotionally ambiguous contexts; (3) Integrating these findings, the study revealed a “differentiation between early and late stages” in the emotional processing patterns of adolescents with NSSI. This atypical neural pattern, characterized by early under-differentiation and late over-engagement, provides preliminary electrophysiological evidence consistent with the view that abnormal emotional processing may contribute to decision-making inhibition deficits in NSSI. However, given the lack of direct correlation between ERP emotional modulation and IGT behavioral performance at the individual level, these findings should be interpreted as exploratory and hypothesis-generating, requiring further validation through correlational and longitudinal studies before they can be considered as neural markers for NSSI risk.

## Data Availability

The datasets presented in this study can be found in online repositories. The names of the repository/repositories and accession number(s) can be found in the article/Supplementary material.
